# Understanding phishing discussions on stack overflow and information security stack exchange

**DOI:** 10.1038/s41598-025-33568-5

**Published:** 2025-12-23

**Authors:** Kholoud Althobaiti, Mohammad Tahaei

**Affiliations:** 1 Department of Computer Science, Taif University, Taif, Saudi Arabia; 2 International Computer Science Institute, Berkeley, CA USA

**Keywords:** Phishing, Developer, Stack overflow, Information security, Code security, Qualitative analysis, Stack exchange, Computer science, Information technology

## Abstract

Phishing remains a prevalent cybersecurity threat. Given its impact, it is important to understand how technically skilled users interpret and respond to such threats. This paper examines how developers and security professionals discuss phishing on Stack Overflow (SO) and Information Security (IS) Stack Exchange in order to understand their concerns, pain points, and investigative practices. We qualitatively analyzed 140 phishing-related questions (60 from SO and 80 from IS) using inductive open coding and developed the Developer Phishing Engagement Framework, which organizes developer activities into four layers: prevention, detection and reporting, mitigation, and planning. Across the two platforms, we find complementary emphases: SO posts focus on implementation hurdles, false positives, and the usability of defenses, whereas IS posts concentrate on post-incident analysis, impact, and ethical considerations around phishing simulations. Developers demonstrate a strong threat mindset but still face workflow friction caused by inconsistent organizational practices, opaque anti-phishing tools, and security measures that conflict with legitimate workflows. Our findings contribute a developer-centered view of phishing that complements existing user-focused models and provides guidance for designing more realistic anti-phishing tools, training, and organizational policies.

## Introduction

Phishing has been recognized as one of the most prevalent cyberattacks, with many incidents traced back to emails or messages designed to deceive users into revealing sensitive information. More specifically, phishing remains one of the leading causes of breaches according to the latest Data Breach Investigation Report^1^. Over the years, attackers have continually refined their social-engineering tactics, increasingly adopting more sophisticated and believable techniques^2^. For example, using adversarial GenAI is ranked among top risks^3^, and recent studies show LLM-crafted spear-phishing can rival human attempts, while QR-based phishing is succeeding in real-world settings^4^.

Studies have shown that individuals with a technical background have more awareness of phishing attacks compared to their peers with other backgrounds^5–7^. The process followed by IT experts can be broken down into three steps: understanding the email, finding discrepancies, and, once suspected, investigating the email^8^. The investigation can be simple and straightforward, as phishing emails often have unique characteristics such as poor structure and spelling errors^9,10^; however, a lack of security background can make it difficult for technically skilled users to recognize and identify phishing^6,9,11^. Among those with technical backgrounds, developers represent a key subgroup who frequently seek help from their peers, specifically through online queries about distinguishing phishing attempts^12^. Given the fast-evolving nature of phishing threats, developers often rely on online technical communities such as Stack Overflow for real-time problem-solving and shared experiences. Unlike structured training or cybersecurity certifications, these forums offer immediate access to peer insights, emerging threats, and practical solutions, making them a critical resource for understanding phishing.

Although phishing is widely studied, developers remain understudied-despite being both frequent targets^13^ and central actors in designing and maintaining systems that must resist phishing attempts^14–16^. Recent studies also show that adversaries explicitly target humans involved in software development: for example, social engineering campaigns aimed at stealing PyPI credentials and tricking developers into installing malware on their devices^13^, and more broadly, software supply-chain attacks that rely on tailored phishing and spear-phishing to compromise developers and maintainers^17^. Williams et al. warn that advanced phishing using large language models to craft highly deceitful messages is likely to become a rising threat to the software supply chain by exploiting these human vulnerabilities^17^. Additionally, developers day-to-day technical decisions-such as authentication flows or filtering email and URL shape organizational resilience, yet gaps persist between secure design intentions and actual system behavior^15,18,19^. However, only a small portion of this work examines how developers themselves reason about, investigate, or defend against phishing within their workflows. This study aims to deepen our understanding of developers’ concerns and discussions regarding phishing on Stack Exchange platforms. Our analysis surfaces what developers ask, what issues they encounter, and what reasoning they have. Understanding how technically skilled users reason about phishing on professional communities can help align security guidance, training, and controls with real-world practice.

We examined two widely used Stack Exchange forums: Stack Overflow and the Information Security. Each contains millions of posts covering a wide range of topics, including those related to programming, mobile development, and security^12,20–24^. Our objectives are to characterize phishing-related concerns, tasks, and pain points expressed on Stack Overflow and Information Security, and to compare how these emphases differ across the two platforms. We then propose a framework that organizes developer phishing concerns across the attack life cycle. We qualitatively analyzed 140 phishing-related questions from both platforms through an inductive open coding approach. A qualitative approach is well-suited for this study because phishing, as a social engineering attack, is not just a technical problem but also a psychological and behavioral challenge. By analyzing developers’ discussions, we can uncover underlying concerns, misconceptions, and practical security challenges that may not be evident in quantitative studies^25^. The paper’s contributions are as follows:

- A qualitative synthesis of phishing discussions by technical users on SO/IS, including implementation issues, detection and reporting practices, and post-incident analysis.
- A cross-platform contrast highlighting how concerns differ across communities (SO: implementation challenges and false positives; IS: impact assessment and tracing).
- The Developer Phishing Engagement Framework (DPEF), which characterizes developer concerns across prevention, detection/reporting, mitigation, and forward planning.

Our analysis reveals several categories, as evidenced by discussions on SO and IS platforms. We propose the Developer Phishing Engagement Framework (DPEF), a four-layer lens we use to organize and interpret all results in this paper: L1 Prevention: (pre-attack hardening and safe-practice design), L2 Detection & Reporting (in-the-moment legitimacy checks and reporting paths), L3 Mitigation (post-attack containment and recovery), and L4 Planning (tests, ethics, and investment choices). DPEF is derived from our qualitative coding of SO/IS posts and reflects how developers and security practitioners actually talk about phishing. More over, our findings reveal an interesting contrast between the two platforms. Stack Overflow posts focus on code-level concerns, such as vulnerabilities, risky legitimate practices, and false positives that misclassify safe code or trusted sites. Information Security discussions emphasize detection, reporting, and the broader consequences of phishing, reflecting a more post-incident perspective. Both communities show a similar interest in technical investigations and the tracing of attacks. Overall, SO highlights practical, workflow-embedded defenses, while IS centers on strategic analysis and impact.

The remainder of this paper is organized as follows: The related work section provides an overview of existing research on the phishing life cycle, sources of advice, and Stack Exchange forums. The methodology section details our data collection process, ethical considerations, and qualitative analysis approach. The results section presents our thematic findings, including phishing prevention, detection, and reporting, mitigation, and planning. Finally, the discussion and conclusion sections explore the implications of our findings for developer-focused anti-phishing tools and training programs, and summarize the study’s contributions.

## Related work

In this section, we review related work that informs our study of phishing discussions on Stack Overflow and Information Security. We first summarize phishing life-cycle models and technical attack vectors and discuss existing phishing frameworks. We then discuss where users and practitioners obtain security advice, with a focus on online forums. We also describe prior work on security and privacy discussions within the Stack Exchange network.

### Phishing life cycle

Phishing is a scalable act of deception whereby impersonation is used to obtain information from a target^26^. Based on the work of Shaikh, Shabut, and Hossain^27^, phishing attacks involve five stages: planning and setup, phishing, break-in, data collection, and breakout.

Planning the attack requires gathering details on the selected victims. It also involves choosing the medium for deploying the attack, such as emails, smishing (SMS-based phishing), vishing (voice-based phishing), quishing (QR-based phishing), or websites^14,28,29^. Malicious websites are often used in conjunction with other vectors, such as email, as users typically visit them by clicking on malicious URLs.

The second stage of the attack is the actual phishing. Attackers employ a variety of technical approaches to deploy their phishing attempts. For instance, they may send general phishing emails to a large group of people or engage in spear phishing, which targets specific individuals with content that is relevant to them^11^. In each technique, the attacker typically spoofs a reputable authority and uses a variety of topics that shift over time^30^. Cross-site scripting (XSS) is another technique attackers use to inject malicious code into a vulnerable website; it can serve as an initial step in phishing, with the injected code redirecting users to a phishing webpage^28^. To counter these delivery techniques, a large body of work has focused on automated phishing detection. Ayeni et al. systematically review these approaches and organize them by communication channel, highlighting URL- and lexical-based filters, content and header analysis for emails, and visual similarity and DOM-based checks for phishing websites, as well as machine-learning and deep-learning models that combine multiple features^31^. These technical defenses aim to identify and block phishing artifacts before users interact with them.

The attacker succeeds when the target interacts with the lure-by clicking a malicious link, downloading an attachment, or voluntarily submitting sensitive information such as credentials, addresses, or banking details. Any of these activities can allow the attacker to gain access to accounts, steal money, or initiate other attacks, such as sending spear-phishing emails from the compromised accounts^32^.

After a successful phishing attack, the attacker removes all evidence of the attack and any information that could point to them. This step is critical because the attacker seeks to keep the operation active while avoiding detection by security filters. Because traces are fragile, prior work recommends starting the response with forensics^33^, emphasizing freezing evidence, reconstructing the timeline from lure and click to authentication, persistence, exfiltration, and scoping affected accounts, sessions, and data^33–35^.

While the above life-cycle emphasizes technical stages and infrastructure, phishing is fundamentally a socio-technical problem in which human cognition, behavior, and organizational context play a central role. Human factors research on phishing is increasingly multi-modal, but still highly fragmented across different experimental designs, measures, and attack types^36^. Previous work reveals a strong focus on psychological determinants of susceptibility, user-centric countermeasures, and AI-enabled defenses^37,38^.

Together, these studies show that understanding the phishing life-cycle requires considering both the technical attack surface and the human processes through which people perceive, learn about, and respond to phishing.

### Phishing frameworks and life-cycle models

Prior research on phishing and social engineering has proposed a range of conceptual frameworks and life-cycle models, typically organized around the attacker’s steps or the end-user’s decision process. For example, surveys and systematic reviews structure the phishing landscape in terms of attack types, delivery channels, and technical detection pipelines^37,39,40^. Other work has proposed explicit frameworks and taxonomies for human factors in phishing and social engineering^41^, including the Suspicion, Cognition, and Automaticity Model of phishing susceptibility^42^, and taxonomies of social-engineering attacks that foreground human aspects such as trust, socio-emotional state, and awareness^43^. These frameworks provide important abstractions for understanding phishing at a high level, but they either treat developers as generic employees or do not model developer activities explicitly. Studies of security discussions on Q&A platforms show that developers use these spaces to negotiate security responsibilities and make sense of complex issues^44,45^, yet this work has not been synthesized into a structured view of how developers engage with phishing specifically.

Our proposed DPEF framework organizes our findings about how developers and security practitioners discuss phishing. DPEF aligns developer activities with four layers of organizational defense: prevention, detection and reporting, mitigation (post-attack), and planning. Rather than starting from an abstract attack model, the framework is grounded in inductive coding of in-the-wild questions and answers, and captures the concrete situations where developers become involved in phishing-related work. To our knowledge, DPEF is the first framework that organizes phishing-related developer work as observed in SO/IS discussions, rather than abstracting from end-user or attacker-centric models.

### Security advice and online information sources

In general, when users need technical advice, they often turn to a variety of formal and informal sources, which can include friends, family, colleagues, acquaintances, or technical support in the workplace^46^. Due to the diverse nature of user needs and preferences, the majority of users typically consult several sources of advice. Nevertheless, posting questions on online platforms is the second most preferred method for many users, such as asking on their social networks or turning to forums. Adults with higher-level skills tend to engage in such channels more than others^46^. Among professionals, the workplace can be a source of security know-how as engaging company developers in security activities and tooling shape how developers seek and apply advice^47^.

When seeking security-based advice, the choice of information sources can vary based on the user’s profile and expertise. Tech-savvy individuals often turn to their workplace peers for guidance and tend to develop necessary skills through learning from their own negative experiences^48^. In contrast, average users, lacking similar technical expertise, depend on a wider range of sources for security advice. These sources commonly encompass family, friends, online resources, service providers, and IT professionals^48,49^.

Online forums have become one of the most extensively researched sources of advice and information in this context, especially for developers, who usually turn to forums to solve some practical security hurdles^50^, especially since most of the participants in these security conversations are not formal security experts, but generalist developers who nonetheless engage seriously with secure coding concerns^45^. Notably, these platforms provide more than mere advice; users frequently seek information, opinions, and general insights from these forums^51,52^.

One of the most common topics of security-related discussions in these online forums centers on issues about phishing and malware^12,53^. For instance, questions about phishing on Yahoo Answers often concern the identification of phishing messages and the determination of their authenticity.

### Stack exchange forums

Stack Exchange^54^ is a prominent network of question-and-answer websites that cater to a variety of topics, including programming, security, mathematics, and more. The ability for users to remain anonymous by using pseudonyms is a key feature of Stack Exchange that can foster a more open environment for discussing sensitive security questions^55^. This anonymity can be particularly important in the security domain, where discussing vulnerabilities or incidents may carry a risk if tied to one’s real identity.

Within the Stack Exchange network, Stack Overflow and the Information Security site are particularly well-known. Stack Overflow is highly regarded for its technical community and is one of the most frequented online forums by those with a technical background. It hosts a vast array of security-related questions, covering topics such as web security, mobile security, software security, system security, cryptography, and code vulnerability^21,56^. The platform is widely used by developers, with the 2024 Stack Overflow Developer Survey^57^ showing that a large percentage of developers rely on it for learning and troubleshooting. Lopez et al. find that developers’ discussions on Stack Overflow frequently provide highly tailored assistance, link specific language and library features to concrete security problems, and situate their advice within the broader security landscape^45^. Following prior work, we treat the users of these platforms as developers and security professionals, even though authorship status cannot be fully verified on pseudonymous forums. Interestingly, previous studies have shown that Stack Overflow code is reused at scale and evolves, indicating that developers usually rely on such forums to solve practical issues or find functioning code^58^. Additionally, Stack Overflow’s structured tagging system makes it easier to filter and collect relevant discussions compared to less organized platforms.

Similarly, the Information Security site on Stack Exchange^59^ is dedicated to more specialized security topics to discuss various security-related issues, including social engineering, network security, incident response, and more. It attracts a community interested in detailed and expert-level discussions. The users of Security Stack Exchange include a diverse range of individuals, with top contributors primarily working as developers, security professionals, or penetration testers^60^. Similar to Stack Overflow, studies in Information Security have examined the security challenges developers face and explored the role of security champions in addressing these issues^60,61^. Privacy-related questions also find a place on these platforms, addressing issues such as privacy policies, privacy concerns, and access control^22^.

In summary, the extensive information available on Stack Exchange platforms makes them valuable resources for developers seeking to understand and address security-related issues, including phishing. While previous studies have examined developers’ general security questions or focused on specific security areas, our study builds on this work by analyzing phishing-focused discussions across SO and IS Stack Exchange and situating the emerging themes within the broader lifecycle using the Developer Phishing Engagement Framework (DPEF).

## Methodology

**Fig. 1:**
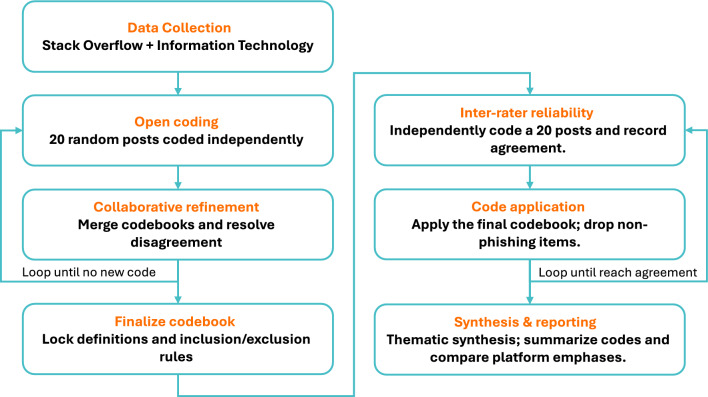
Method workflow for qualitative coding of phishing-related questions from Stack Overflow and Information Security.

We qualitatively analyzed a random sample of posted questions sourced from two commonly used developer forums, which serve as platforms for knowledge exchange among programmers and security professionals. In the following sections, we elaborate on the data collection process, analysis methodology, and study ethics. The methodology block diagram is shown in Fig. 1.

### Data collection

To answer our research questions, we collected phishing-related questions from Information Technology Security^62^ and Stack Overflow^63^, using the SQL queries provided below. Initially, we attempted to use various keywords such as “fraud,” “scam,” “spoof,” and “trick.” However, we encountered a significant number of irrelevant posts that were difficult to exclude. After several experiments with various keywords, we determined that focusing on posts containing the morpheme “phish” in the tags, body, or title would serve as a reliable indicator for identifying questions related to phishing. Data collection took place on July 14, 2023.

SELECT * FROM Posts AS p WHERE p.tags LIKE '%phish%' AND p.PostTypeId = 1 -Questions

SELECT * FROM Posts AS p WHERE p.title LIKE '%phish%' AND p.PostTypeId = 1 -Questions

SELECT * FROM Posts AS p WHERE p.body LIKE '%phish%' AND p.PostTypeId = 1 -Questions

Our quesries resulted in an initial corpus of 1,258 posts (325 from Stack Overflow and 933 from Information Security), we selected a random sample of 140 posts for qualitative analysis. These 140 posts formed the full dataset for the study.

### Data analysis

We employed an inductive open coding approach for data analysis^64,65^. Initially, two authors independently coded a random sample of 20 posts from each platform, each creating a unique preliminary codebook. Through a series of collaborative sessions, the authors coded an additional 20 posts per session, merging their codebooks and resolving discrepancies through discussion. We repeated this cycle (independent coding $\longrightarrow$ comparison $\longrightarrow$ refinement) until no new codes emerged. In total, 80 posts were collaboratively analyzed during this phase, indicating code saturation and resulting in a stable, consensus-based codebook.

Once the codebook was finalized, we conducted an inter-rater agreement check to ensure that both authors were applying the agreed-upon definitions consistently before proceeding with the full dataset. The two authors independently applied the final codebook to a new set of 20 posts drawn from the 140 sampled posts. For this alignment check, we treated each post as a single coding unit and counted an agreement when both coders assigned the same set of codes to that post. Percent agreement was computed as the number of posts with identical coding divided by the total number of posts in the subset, multiplied by 100, yielding 78% agreement. We interpret this as solid coder consistency^66^. Although percent agreement does not adjust for chance and has known limitations^67^, it is widely used in qualitative research as a pragmatic check on coder alignment, especially when combined with consensus discussions, as in our study. After reviewing and discussing the remaining disagreements, we proceeded to apply the finalized codebook to the full set of 140 posts.

**Fig. 2:**
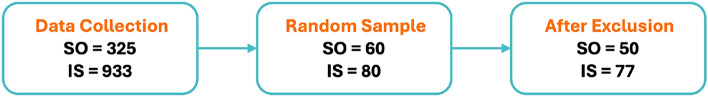
Filtering and selection of posts during qualitative analysis.

During the full-application phase of coding, 13 posts were excluded as false positives because the term “phish” appeared only within code snippets or unrelated technical content. The final analytic dataset, therefore, consisted of 127 posts (50 from Stack Overflow and 77 from Information Security)-see Fig. 2. No framework was pre-imposed during coding. After coding all 127 posts, we grouped recurring defender concerns into the four layers of the Developer Phishing Engagement Framework (DPEF), which we use only to structure the reporting and interpretation of results, keeping the analysis inductive and grounded in the data.

**Fig. 3:**
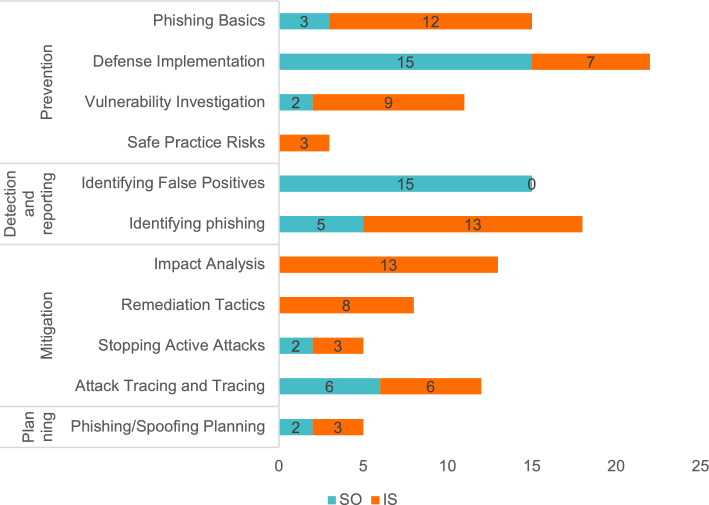
Summary of the codes from SO and Information Security Exchange (IS).

The findings discussed below are derived from this analysis and the finalized codebook. In light of our study’s qualitative nature, we chose not to quantify occurrences in the results section. Instead, we use qualitative descriptors such as “few,” “some,” “several,” “many,” and “all” to avoid over generalization^68^. For readers interested in the specific frequencies, we have included a summary of code occurrences for each platform in Fig. 3.

### Study ethics

Our research complies with the terms of service and privacy policies established by Stack Exchange. Content on the network is publicly accessible and shared under a Creative Commons license, and Stack Exchange encourages scholars to use its data for research purposes^69,70^. All queries were executed through the Stack Exchange Data^54^.

While the public nature of Stack Exchange data aligns with ethical guidelines for internet research, prior work shows that the public status of online content does not automatically align with participants’ expectations. Fiesler and Proferes^71^ found that users of open platforms such as Twitter often do not anticipate that their posts will be used in academic research, and their comfort depends heavily on context, the sensitivity of the topic, and whether their content can be traced back to them. A related synthesis of internet research ethics highlights similar tensions, emphasizing that even on technical or pseudonymous platforms, researchers must “remember the human” behind each post and consider how quoting or recontextualizing content may expose individuals in ways they did not predict^72^.

Guided by these insights, we treat Stack Exchange contributions as reflections of developer experiences rather than impersonal data points. We avoided collecting any profile or demographic information, and we do not link to user pages or include verbatim text that could make a post easily discoverable. Because technical platforms can expose professional identity or past security incidents, we paraphrased or lightly de-identified some example quotes to minimize traceability while preserving meaning. This approach aligns with best practices recommended in prior empirical studies on participant expectations and ethical use of public online data. By sharing our SQL queries and documenting our methods transparently, we seek to balance openness with a deliberate effort to minimize potential harm or unwanted exposure to contributors.

## Results

**Fig. 4:**
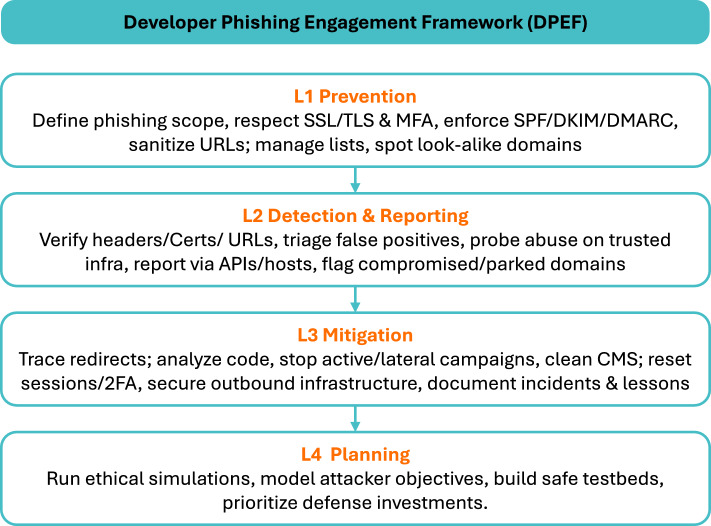
Four-layer Developer Phishing Engagement Framework (DPEF).

We organize the findings with our proposed Developer Phishing Engagement Framework (DPEF): (L1) Prevention, (L2) Detection & Reporting, (L3) Mitigation (post-attack), and (L4) Planning. The DPEF framework is shown in Fig. 4. When reporting the results, we use the abbreviation ’IS’ to indicate posts from Information Security, whereas ’SO’ denotes questions from Stack Overflow.

### L1 - prevention

Preventing phishing involves individuals or organizations applying proactive measures to stop future attacks before they reach inboxes. We have categorized this category into four sub-categories:

#### Phishing basics

This sub-category covers general non-technical topics on protecting users or organizations from phishing attacks. One question directly asks about the meaning of phishing and which attacks fall into the category of phishing and which do not (IS51). While the definition may seem straightforward, it becomes more complex when phishing is combined with other attacks, making it difficult to recognize^73^. Even if users can differentiate them, they also want to understand how phishing is used to initiate other attacks, such as ransomware and Cross-Site Scripting (XSS) (IS53, IS48).

From an organizational perspective, questions generally revolve around strategies for enhancing the security of web applications and online banking platforms (SO46, IS44). Examples of such inquiries include, “*What kinds of protections can an online banking site use to defend against phishing,... what is the reasoning behind why those protections are effective?*” Some queries extend their focus to gaining deeper insights into the fate of phishing and malicious websites post-flagging by filters like Google Safe Browsing (IS47). Additionally, there is interest in understanding how SSL certificates contribute to verifying an organization’s domain ownership and establishing trustworthiness (SO48, IS49). While these questions pertain to organizations, it is unclear whether the inquirers seek information for their own organizations or aim to grasp the broader landscape.

Other questions explore the importance of multi-level authentication (IS50), “*The only benefit I can think of is that if someone forgets to log out, or if another person learns the main username and password, that person still wouldn’t be able to take any action*” This advantage was also questioned by another asker who thinks an attacker gained access to their account even with these measures, while they assured that they did not give away their passwords voluntarily through phishing (IS46). People were curious about SSL certificates. One person asked why browsers show scary warnings about SSL (e.g., expired or untrusted certificate). They wondered if users might think it means phishing, claiming that having an expired SSL is better than having none at all (SO48). Another person asked about the importance of SSL for detecting phishing when even phishing websites can use encryption (IS52). While the first asker’s concern about users’ misunderstanding was confirmed in previous research^74,75^, it is interesting to note that developers are discouraged from using such technology to avoid scaring off their users from their websites and because they are not aware of its importance in detecting other cyber attacks, such as the Man in the Middle attack.

Additionally, there’s curiosity about how anti-phishing solutions protect users’ privacy (IS55). Another question revolves around how email servers handle emails lacking SPF/DKIM/DMARC. The user speculates whether servers check for MX and score incoming emails accordingly, posing the question, “*Surely the filters also look at the MX records and assign a score to incoming mail based on that, right?*” (IS45) Another question seeks clarification for dataset hashing (IS58), “*Why are we hashing this at all? What security issue is that actually addressing? From my perspective, hashing only reduces how useful the list can be.*”

#### Defense implementation

This category revolves around key aspects related to phishing and security measures. It includes questions about accuracy, effectiveness, reliability, and practical guidance in preventing phishing. Each question addresses a specific technical challenge, highlighting the diverse obstacles faced by developers and researchers in the phishing field.

Questions touch on technical challenges in implementing anti-phishing solutions (SO1, SO6, SO7, SO10, SO49, SO52, SO50, SO51), such as integrating APIs, “*I’ve searched all over the web and still haven’t found a working example - not even in the official developer guide,*” handling URL redirections with Curl, “*curl doesn’t appear to follow every redirect. I’m trying to figure out what the final target URL in the redirect chain is,*” extracting, processing, and classifying publicly available datasets, along with looking for up-to-date datasets.

Other questions focus on improving efficiency (SO4, SO5, IS35, SO53, IS36) or the usability of implemented code (SO9, IS2, IS3), like adding a feature to detect Homoglyph attacks, whitelisting commonly used websites, or enhancing security measures without compromising user freedom. Some questions provide detailed automatic filtering scenarios, such as accepting signed, encrypted, and whitelisted emails, while others seek a way to check if the company domain is being used in phishing.

Certain questions seek solutions for specific problems (IS16, SO51). For instance, one user sought a prevention method to forward unknown callers to a specific number by checking against a whitelist.

Some other questions do not directly address phishing but focus on authenticating communication parties to prevent phishing attacks (SO2, SO3, SO8, IS34). For example, users look for alternative solutions for TLS certificates to allow bankers to prove their identity without relying on fancy logos.

#### Vulnerability investigation

This category reflects a concern that is becoming increasingly important in the realm of software development and cybersecurity. It revolves around ensuring that one’s code is secure and does not inadvertently become a tool for malicious activities, particularly phishing. It involves questioning vulnerabilities in both the code that developers wrote themselves and the currently used security practices.

The most common code vulnerability is unauthorized URL redirection, where attackers rely on users’ trust in a legitimate website to redirect them to a phishing website (IS17, IS18, SO38). One form of unauthorized redirection is URL cloaking, a technique used to hide the actual destination of a URL by presenting a different final webpage URL based on the type of users^76^. One of the questions was asking why Facebook shows a preview of the final webpage while the attacker can change it, “*If the original site later removes that redirect, your old link can still look legitimate (depending on the site you first pointed to), but in reality it might now lead to a fake phishing site.*” Another form of unauthorized redirection is when the attacker can manipulate the legitimate website URL by adding a phishing URL in the URL query. Once the user clicks on the legitimate link, they will be redirected to the phishing one (IS18, SO38), with one of the users asking how a developer can remove such vulnerability, “*Is there any free tool or service that can analyze my source code for security flaws? If not, what alternatives do I have?*”

There are security concerns in OAuth 2.0 and OpenID Connect related to installed apps (SO45, IS41). OAuth 2.0 and OpenID Connect are protocols commonly used for authentication and authorization in web and mobile apps. While effective, they do not protect users against phishing attacks^77^. One user questioned the exposure of the client ID in OAuth 2.0 PKCE for installed apps, expressing concerns about attackers creating fake apps, deceiving users into granting access, and obtaining unauthorized refresh tokens, “*I don’t see any real barrier that would prevent an attacker from impersonating gcloud and using that to get into a user’s GCP environment.*” In OpenID Connect, a similar concern arises about malicious apps stealing client IDs through impersonation of official apps.

Other questions encompass various concerns in cybersecurity. The first inquiry into distinguishing operating system-issued password prompts from application-delivered prompts during package installations, especially from untrustworthy sources (IS37). The second question raises potential security issues with a checkout overlay, pondering whether the service employs measures to prevent malicious websites from mimicking their overlay (IS38). The third question revolves around cryptographic security tokens following the FIDO U2F specification, exploring its strengths and weaknesses against phishing (IS39), and the effectiveness of two-factor authentication (2FA) (IS43). The fourth question explores the effectiveness of the warning message “*THIS EMAIL IS FROM AN EXTERNAL SENDER*” in preventing phishing attacks, considering potential banner blindness (IS40). The final inquiry concerns verifying the legitimacy of embedded login pages in mobile apps, particularly in the context of social logins, where consumers face challenges in confirming the authenticity of such pages without standard browser visibility (IS42).

Most of the questions are valid security concerns that imply developers pay attention to various aspects of application development and online interactions, signifying the critical importance of robust security awareness^78^.

#### Safe practice risks

This category discusses the possibility that every day, seemingly legitimate practices could inadvertently expose individuals or organizations to phishing risks. This concern is rooted in the realization that regular activities and digital habits, while benign in intent, can be exploited by phishers. One university student raised concerns about their university’s Central Authentication Service (CAS). They noticed that all web services use CAS except for one page that asks the user to enter their CAS credentials with a different login page. Thus, they think it may train users to enter their credentials on any website (IS56). The other question is about why big organizations use various email domains in their emails, which makes it harder to decide on the email’s legitimacy, “*the only option is to extensively search for whether the sender’s domain really belongs to the legitimate company?*” and suggested that this may raise difficulty users may face in distinguishing between legitimate and scam websites if such practices become common (IS57, IS21), “*we’re being ’re’-trained to put our trust in domains than the official ones.*” Their inquiries, although limited, reflect a commitment to understanding, improving, and ensuring the robustness of cybersecurity measures in the ever-evolving digital landscape.

### L2 - detection and reporting

This category focuses on the detection of phishing attacks, exploring questions about both manual and automatic detection and tackling the challenge of false positives in the detection of legitimate practices.

#### Identifying and reporting phishing

This category encompasses scenarios where users are uncertain about the legitimacy of various digital communications, including emails, URLs, or phone calls, and seek guidance on identifying and reporting potential phishing attempts.

While this type of question was observed in previous research, we noted that the suspected communications shared on these platforms are more challenging to detect compared to what average users suspect^12^. The suspected communications in this category are either sophisticated phishing attempts or legitimate communications with misleading practices. These questions involve inquiries about emails coming from a legitimate IP address but with an unknown email address for the recipient, “*I’m not sure why this email keeps showing up, but when I checked the requests, they were originating from an Amazon AWS IP address.*” (SO40). In other questions, the email address was trusted, but the email body had many signs of suspicion (SO41, IS26), such as a suspicious URL domain issuer, “*The URLs point to an unfamiliar domain whose TLS certificate is issued to a different organization than paypal.com, and one of those links delivers a PDF file.*” As discussed above, this issue may result from companies starting to use dedicated domains or subdomains for their services other than the official known domains, raising developers’ suspicions of subdomains hosted under trusted domains (IS22, IS23, IS29), “*Can an attacker register a subdomain of a known site for phishing?*” An interesting but odd question was about visiting GitHub from the Google search engine, but after a failed login, the user realized the URL in the address bar was not GitHub’s URL and was wondering if this was phishing or something else (IS25).

Other posts ask about more technical methods to investigate the legitimacy of phishing emails (IS33), “*Is there any technical method to identify these kinds of fake messages?*”, emails coming from spoofed email addresses (IS24), “*Why is this still so fundamentally flawed?... When I visit* https://www.apple.com*in a browser, I have a solid assurance, but if I get a message claiming to be from support@apple.com, there’s basically no comparable guarantee.*” Also, whether a pop-up window is suspicious or not as a result of spoofing or a security vulnerability (IS30, IS19).

For phishing reporting, one user asked about reporting phishing automatically (SO44), “*I’d like to use the Gmail API to report an email as phishing*” We noticed that developers’ questions on reporting differ from the common sense of phishing reporting. They seek ways to report phishing emails from compromised legitimate accounts (IS20), abandoned compromised domains (SO43, IS32), phishing websites hosted on legitimate web services (SO42), “*Does anyone know the proper way to report a phishing website that’s hosted on Heroku?*”, vulnerabilities in web hosting services (SO43, IS31), “*Do you know a kind of security vulnerability in WordPress whereby fraudsters send out fake banking emails for phishing?,*” or malicious ads on well-known websites (IS27), “*I’ve already contacted the companies about these ads, but they basically ignore my emails.*”

#### Identifying false positives

This category addresses the common challenge in cybersecurity and anti-phishing measures: false positives. A false positive occurs when legitimate systems, websites, emails, or activities are incorrectly identified or blocked as malicious or phishing attempts. We observed uncertainty among developers about how anti-phishing tools work, which may also indicate a lack of awareness of email and website best practices.

Most questions relate to websites being blocked by phishing detection systems (SO24, SO25, SO28, SO23, SO29, SO27). Some developers are unsure what might trigger a phishing alert, while others are aware of the cause but seek solutions to bypass it without engaging in illegal activities, as one developer stated, “*Just to be clear, this is entirely legitimate - it’s simply an interface wrapper around a webcam.*” Examples of such activities include developing an invisible frame, cloning an Instagram page, changing CNAME records, or using JSON data from a blocked website.

Other questions concern legitimate emails ending up in the junk folder or being blocked by specific mail clients (SO27, SO30, SO31, SO32, SO34). In many cases, the reasons for the blocks are unknown to the developers, “*Our emails sent via GoDaddy/Mailgun are being marked as phishing - is this possibly linked to the DMARC configuration?*” Sometimes, emails are blocked by one mail client but work well with others, “*Certain mail clients, especially Office 365, are classifying our messages as potential phishing,*” “*I’m having trouble receiving emails for some domains. I suspect spam filtering is the cause, so I’ve already verified that the headers are configured properly.*”,“*The Emails are HTML and contain a link in the body, and whenever they’re sent to Gmail addresses, they end up directly in the spam folder.*”

Additional questions involve code not working properly, with users suspecting that anti-phishing tools are the cause (SO33, SO37), a Facebook-like button not functioning due to a red flag (SO35), and cURL proxy code being red-flagged by Kaspersky (SO36).

### L3 - mitigation (post-attack)

After successful phishing attacks, victims typically explore post-phishing activities. We categorize these activities into technical investigation and tracing, ongoing attack response, individual remediation efforts, and understanding attack consequences for a comprehensive guide.

#### Attack tracing and investigation

This category focuses on the actions taken by developers to analyze, trace, and understand phishing attacks using specialized tools and security analysis methods.

Questions involve a detailed examination of various aspects of phishing after confirming their malicious nature (SO55, SO56, SO59, IS64, SO57, SO58, IS63, IS71, IS70, IS72). Examples include analyzing PowerShell code to understand its full functionality, understanding the behavior of a phishing URL and redirection in various browsers, the reasons for using WebAuthn instead of plainly asking for credentials, understanding the functionality of JavaScript phishing code, and creating a sandbox for investigating email attachments: “*I want to set up a sandboxed environment where I can inspect this type of email, since I’ll probably need to do this repeatedly rather than just once.*” Interestingly, one case involved an email that was not phishing, but the user decided this after a thorough investigation (IS65).

While askers seek help, the reasons for their investigations are not always clear: “*I just need a breakdown of what this code does,*” “*I’m trying to understand how this exactly works*,” even after considerable research: “*I have done a ton more research since I first posted this question and I think I had a few terms goofed up as well.*”

One program was prohibited due to its dangerous nature after a phishing attack, and the asker was looking for a way to replicate its functionality (SO39).

#### Stopping active attacks

This category encapsulates scenarios where users or organizations attempt to mitigate ongoing phishing attacks. It highlights the challenges and limitations faced in real-time cyber defense, particularly against phishing.

Users seek guidance on several situations, such as failing to stop lateral phishing, where attackers gain unauthorized access to a victim’s account and use it to send spear phishing (IS7, IS54), with some emails passing SPF, DMARC, DKIM, and ARC (IS8). Others seek advice on stopping phishing emails originating from their web applications (SO21) or preventing the uploading of phishing websites on a shared server they manage (SO22).

#### Remediation tactics

This category pertains to the actions and measures individuals take in response to phishing attacks.

Questions address practical steps victims can take to mitigate damage, secure their information, and seek redress. This includes additional steps when a WordPress site contains malicious payloads (iframe and RIG exploit) (IS70), actions after sharing personal information (IS10, IS77), advice after opening a phishing email and running antivirus scans (IS12), and steps after clicking on links and running several antivirus scans (IS15).

Other questions concern the credibility of scenarios where OTPs for online transactions can be bypassed (IS9) or general security guidance on logging off all sessions after changing a password (IS14).

Some askers are already cautious, such as one who usually uses virtual machines to surf the web and open suspicious emails, but made a mistake this time. The results of this category align with other studies on general security and privacy questions^12^.

#### Impact analysis

This category focuses on understanding the broader implications and motivations behind phishing attacks, rather than the technical investigation of these motives. It is crucial for comprehending the multifaceted nature of phishing threats and is primarily discussed on Information Security Exchange, not Stack Overflow.

Questions involve understanding risks beyond monetary loss from personal data exposure (IS62, IS76), why attackers use multiple emails (IS68), why scammers use complex techniques instead of simpler phishing forms (IS69), and whether an attacker can access a computer on the same network as a compromised one (IS73).

Other inquiries include the motives behind deceptive emails linking to genuine sites (IS66), what attackers gain from emails without hyperlinks or attachments (IS67), whether attackers can compromise devices through a phone call or by visiting a phishing website (IS74), and the best actions when receiving unsolicited OTPs (IS28).

Some askers are unsure if they have been attacked by phishing or another method, such as receiving an email from a friend with a suspicious attachment (IS79) or a phishing email about a large PayPal deposit (IS80).

### L4 - planning

This category captures forward-looking work: planning awareness tests and demos, clarifying ethics/legal boundaries, and setting up safe local testbeds for phishing scenarios. Askers turn lessons into lightweight playbooks with simple metrics so fixes become repeatable guardrails.

#### Spoofing/phishing planning

This category covers topics related to the techniques and strategies for executing spoofing or phishing attacks, with a careful examination of ethical and legal boundaries.

The intention behind these inquiries was explicitly mentioned in some posts. For instance, one user was planning a real attack for a TV show, while another was organizing a user awareness test.

One user inquired about the feasibility of a phishing attack designed to harvest Facebook credentials and bypass two-factor authentication (2FA) (IS59). Credential harvesting was also the focus of another question, which delved into the technicalities of using cURL to redirect users to a legitimate webpage after a successful attack (SO54). Another individual was tasked with conducting an attack on a TV show, stating, “*I’ve been given the chance to carry out an actual hacking demonstration for a television program.*” While their plan involved initiating a phishing attack as a precursor to more damaging cyberattacks, they sought advice on ideas for additional attacks that could be visualized (IS60). In a different scenario, a user conducting a user awareness test pondered the ethical implications of spoofing well-known brands and storing user data without explicit consent (IS61). Interestingly, one user was attempting to run a phishing attack on localhost, but their Chrome/Firefox browsers were not validating the legitimacy of the page. Consequently, the user sought guidance on how to make the browser validate the webpage for testing purposes (SO60).

## Discussion

Our analysis of the posts on the two platforms, Stack Overflow and Information Security Exchange, resulted in diverse and interesting categories viewed through DPEF. Recent phishing research reinforces the value of lifecycle-based framings. Contemporary reviews show that phishing attacks evolve through distinct stages, from initial infrastructure setup to long-term exploitation^14,79^. Similarly, organization-oriented analyses highlight that defenses map onto different stages of the attack cycle rather than a single point in time^38^. In our study, developers’ concerns cluster into pre-attack hardening (L1), in-the-moment judgment and reporting paths (L2), concrete containment/cleanup after exposure (L3), and scenario design and ethics (L4). This lens clarifies where friction arises, such as branding inconsistencies, unclear channel signals, false positives, fragmented reporting, and where organizations can intervene. Using our proposed framework can help capture developers’ holistic pain points and design solutions that would help in each stage. This framework can be replicated to study ongoing, community-based learning on any platform and understand any evolving issues and problems^80^.

### Complementary perspectives across SO and IS

Overall, our results showed that the knowledge bases on these platforms are complementary. Our observations align with recent empirical work showing that security-focused developer discussions on Stack Overflow often reveal configuration struggles, misalignments in security reasoning, and gaps between official documentation and real-world practice^44,81,82^. On Stack Overflow, we found more technical questions about how to prevent, detect, and deal with phishing attacks. These questions often focus on writing code or implementing security measures in software. In contrast, on Information Security, discussions go beyond just the technical side. They dive into the bigger picture of phishing, looking at things like why attackers use certain tactics, how phishing affects people and businesses, and the ethical considerations of conducting phishing tests. This beneficial relationship implies that the two communities provide a wide range of perspectives on phishing, from the practical to the theoretical, offering a comprehensive overview of concerns. This distinction is crucial because it highlights how developers engage with phishing not only as a security risk but also as a challenge embedded within their workflow.

Given that SO skews toward hands-on implementation “how-to” while IS offers a broader perspective on motives, impact, and ethics “why/when”, the practical implication is a two-track support model: SO-style materials paired with IS-style. Reprioritize tooling to fix SO pain points while using IS insights to harden organizational policy and incident playbooks, and build bridges between the communities so code fixes are not divorced from security rationale.

### Developers’ concerns compared to average users

We observed that developers’ questions tend to be a combination of technical expertise, specificity, and proactive engagement in incident response, distinguishing them from the inquiries of average users that focus on personal risk^8,9^, such as the legitimacy of emails and questions on phishing mitigation and remediation^83^. Technical users focus on solving problems they face when building things, like websites or apps, to make their projects secure and find ways to spot and stop phishing attacks. For example, they might ask about how to make sure a website is safe from hackers. Developers must balance phishing prevention with usability, ensuring their security implementations do not hinder legitimate operations.

For phishing detection, average users usually follow several cues such as looking at the from address, URLs, grammatical and spelling mistakes, and discrepancies, and familiarity with email topic and details^83–87^, whereas technical users use multi-step reasoning processes involving technical evidence such as headers, certificate chains, sandboxing, and URL decomposition^8^. While there are similarities between experts and average users, technical users search for more technical evidence in suspected emails, such as hovering over links, opening links in a virtual box, opening attachments in a sandbox, opening up the email header, and running the attachments through antivirus detectors^8^. We observed that our results are in alignment with previous studies on technical users, since the askers usually ask their questions after going through these steps, highlighting that developers only seek assistance with complicated and complex examples of suspicious emails.

The post-attack activities involve securing accounts and reporting phishing. Average users usually report phishing to protect others and ask questions about what steps they should take next after clarifying their actions, such as clicking on links or opening emails^83^. Our analysis showed that developers are actively engaged in post-attack activities with a wider focus on other aspects, such as attack tracing and impact analysis. They seek assistance in understanding and addressing the aftermath of phishing attacks. While developers also report phishing^8^, in our case, they also aim to report vulnerabilities and abandoned domains, attempting to fix the problems from the roots, indicating a proactive approach to cybersecurity incident response. This proactive engagement suggests that phishing awareness among developers should not be limited to recognizing fraudulent messages but should extend to understanding how phishing techniques exploit system vulnerabilities. Training programs should incorporate phishing detection into secure coding practices, API security, and authentication mechanisms, ensuring that developers are not just aware of phishing threats but also actively designing against them.

Overall, developers’ phishing-related questions demonstrate a combination of technical expertise, specificity, proactive engagement in incident response, and awareness of ethical and legal considerations, distinguishing them from the inquiries of average users.

### Developers are already in the correct threat mindset

In order to effectively protect systems and software, developers must adopt the proper threat mindset. Similar to findings in other studies^88^, our results suggest that developers on SO and IS are embracing this perspective. The characteristics we observe mirror broader descriptions of the “security mindset” found in recent qualitative interviews with security professionals^89^. Developers’ willingness to question assumptions, think adversarially, and evaluate system interactions reflects the type of holistic reasoning associated with secure practice. Their comprehensive view of systems emphasizes a thorough approach to anticipating potential issues or vulnerabilities in both their own developed systems or software and those provided by others. They recognize that the strength of a unit lies not just in its individual parts but in its collective resilience. This mindset extends to questioning the safety of established practices, demonstrating a proactive defense mechanism essential for combating such devious and evolving attacks. Additionally, their proactive investigation of phishing efforts, regardless of the threat severity, shows dedication to identifying and addressing vulnerabilities^8^.

This mindset also encompasses a focus on the usability of phishing systems for all types of users, involving the design of warnings and considerations for keeping users safe without imposing significant constraints on their freedom. This is particularly important when designing phishing detection tools or email security filters. Developers’ concerns about false positives indicate that phishing detection systems should be flexible enough to minimize disruptions to legitimate workflows. Security tools could integrate more advanced verification methods, such as contextual AI-based detection, to distinguish between genuine and malicious activities, thereby reducing unnecessary alerts while maintaining high detection accuracy.

### Aligning organizational phishing responses with developer needs

Although developers already have the right threat mindset, they struggle with fragmented advice, inconsistent channel signals, and opaque anti-phishing tooling. Our findings highlight a disconnect between structured, top-down organizational policies for managing phishing incidents and the practical, day-to-day challenges developers face. This organizational mismatch echoes findings from recent studies showing that although organizations focus heavily on formal training or policy development, they often overlook the everyday constraints faced by developers managing security-critical workflows^37^. While organizations often rely on formalized remediation protocols, incident response playbooks, and static training modules^90^, developers frequently encounter complex, edge-case scenarios that these standard practices do not adequately address^8,53^. Developers often seek specific, practical advice on platforms like Stack Overflow and Information Security Exchange, not just about procedural steps but for nuanced issues such as handling spoofed domains, automating phishing report processes, and managing security flaws in third-party applications. What makes it a bit concerning is that, aligned with our observation, these questions are tightly coupled with everyday development work rather than abstract best practices^44^.

Even when organizations follow national regulations or adopt established cybersecurity frameworks such as NIST, ISO 27001, or COBIT-frameworks that strengthen governance and compliance^91^-these high-level standards do not always translate into the implementation-level guidance developers need. Prior work emphasizes that phishing defenses must integrate human, organizational, and technical controls^92^; however, our results show that developers often encounter nuanced, edge-case scenarios where formal policies offer little actionable support. As a result, developers frequently turn to Stack Overflow and Information Security Exchange for practical solutions related to handling spoofed domains, tracing attacks, or managing third-party security flaws^44^.

Our study suggests that organizations should shift from traditional, one-size-fits-all training to more dynamic, scenario-based learning that reflects real-world challenges. Developer-focused anti-phishing tools with contextual support-such as recommending remediation steps when encountering security warnings-can bridge the gap between abstract policies and everyday development work. Facilitating internal knowledge sharing among developers can further strengthen organizational resilience by ensuring that practical insights feed back into clearer, more relevant security policies and training materials.

### Awareness is a necessity for developers

Phishing is often the first step in a chain of other cybersecurity attacks. While it is clear that some developers think about security, they struggle with defining and recognizing phishing in some edge cases where developers openly weigh conflicting cues. In other words, awareness alone is insufficient; how people make decisions under uncertainty and what they know about phishing both matter^93^. Developers need to learn not only what phishing is but also how it interacts with and enables other threats. This confusion can lead to security vulnerabilities in systems and software they are building and protecting^88^. Especially that while Stack Overflow can help practitioners understand the threat and solve problems, sometimes it results in insecure outcomes emerging from partial, copy-pasted answers and difficulty reasoning about the implications of configurations^81^.

Developers need to be equipped with tools that may help them investigate suspicious communications without the need for looking for help^74^. The challenge of phishing identification and recognition is not always a matter of lacking information^7^ but also the complex and evolving nature of phishing attacks.

Awareness does not only include learning about phishing as a threat but also learning about how anti-phishing works and the best practices to develop systems that do not clash with these anti-phishing tools, either because of vulnerabilities in these codes or having illegal actions, and also help users deal with them without suspicion.

Although individuals seek guidance from technical users regarding phishing^48,83,94^, our observation indicates that these technical users often turn to their peers for assistance when it comes to helping their family, relatives, and friends. Enhancing awareness not only strengthens developers’ security expertise but also benefits the wider community. Organizations should recognize that developers play a dual role-both as professionals securing software and as informal security advisors within their circles. By equipping them with stronger security knowledge, organizations indirectly improve overall cybersecurity awareness beyond just the workplace.

## Limitations

We encountered a few limitations in our investigation. Firstly, not every user on Stack Overflow is a native English speaker. This means that due to linguistic variations, we could not comprehend some questions completely. Secondly, phishing attacks frequently occur in conjunction with other types of cyberattacks. As a result, it was challenging to determine if users were discussing phishing explicitly or something else. In an attempt to address this, we meticulously selected our search terms to concentrate only on phishing, taking into account the descriptions provided by Stack Overflow users. In addition, qualitative coding is inherently subjective, and different researchers may identify varying categories depending on their interpretations of the data. To mitigate bias, we conducted independent coding, followed by collaborative coding sessions to refine our codebook. Discrepancies were resolved through discussion, ensuring more consistent categories. Additionally, while our study did not analyze developer experience levels or the temporal evolution of discussions, we recognize these as valuable directions for future research.

## Conclusion

We conducted a qualitative analysis of 127 phishing-related questions on Stack Overflow and Information Security Stack Exchange. Our findings indicate that developers inquire about a broad range of topics related to phishing, including general knowledge about phishing, the implementation and debugging of anti-phishing tools, and the mitigation and remediation of phishing attacks. They are aware of the threat and try to act, but gaps remain in shared practices and tooling. To organize these concerns, we proposed the Developer Phishing Engagement Framework (DPEF), which groups them into four layers (L1–L4) and turns them into actionable guidance for research and practice.

## Data Availability

The datasets used and analysed during the current study available from the corresponding author on reasonable request.
